# DPP-4 Inhibitor Attenuates Toxic Effects of Indoxyl Sulfate on Kidney Tubular Cells

**DOI:** 10.1371/journal.pone.0093447

**Published:** 2014-04-22

**Authors:** Wei-Jie Wang, Chen-Hung Chang, Mao-Feng Sun, Sheng-Feng Hsu, Ching-Sung Weng

**Affiliations:** 1 Department of Biomedical Engineering, Chung Yuan Christian University, Chungli, Taiwan; 2 Division of Nephrology, Department of Internal Medicine, Taoyuan General Hospital, Ministry of Healthy and Welfare, Taoyuan, Taiwan; 3 Department of Pharmacy, Taoyuan General Hospital, Ministry of Healthy and Welfare, Taoyuan, Taiwan; 4 School of Chinese Medicine, College of Chinese Medicine, China Medical University, Taichung, Taiwan; 5 Department of Acupuncture, China Medical University Hospital, Taichung, Taiwan; 6 Graduate Institute of Acupuncture Science, College of Chinese Medicine, China Medical University, Taichung, Taiwan; 7 Department of Acupuncture, China Medical University Hospital, Taipei Branch, Taiwan; University of Pecs Medical School, Hungary

## Abstract

Diabetic nephropathy is a common causative factor of chronic kidney disease (CKD). DPP-4 inhibitor has the ability to improve kidney function and renal microvasculature. In the present study, we investigate the deleterious effects of IS on proximal tubular cells and the protective role of DPP-4 inhibitor. Human kidney 2 (HK-2) cells were exposed to IS in the presence or absence of DPP-4 inhibitor. Effects of DPP-4 inhibitor on viability of HK-2 cells were determined by MTT assay. Reactive oxygen species (ROS) production was examined using fluorescent microscopy. Levels of cleaved caspase-3, transforming growth factor-beta (TGF-β), α-smooth muscle actin (α-SMA) and NF-kappaB p65 and phosphorylation of AKT and extracellular signal-regulated kinase (ERK) were detected by immunoblotting. Production of ROS and level of cleaved caspase-3 were increased by IS in a dose-dependent manner. The phosphorylation of AKT and ERK p65 were decreased alongside activation of NF-κB. Expression of TGF-β and α-SMA, were upregulated in IS-treated HK-2 cells. Treatment with DPP-4 inhibitor resulted in a significant increase in cell viability and a decrease of ROS production in IS-treated HK-2 cells. DPP-4 inhibitor restored IS-induced deactivations of AKT and ERK and inhibited activation of NF-κB in IS-treated HK-2 cells. Moreover, DPP-4 inhibitor could also attenuate IS-induced up-regulation of TGF-β and α-SMA expression. These findings suggest that DPP-4 inhibitor possesses anti-apoptotic activity to ameliorate the IS-induced renal damage, which may be partly attributed to regulating ROS/p38MAPK/ERK and PI3K-AKT pathways as well as downstream NF-κB signaling pathway.

## Introduction

Type 2 diabetes is characterized by an increased insulin resistance and poor glucose tolerance in a conjunction with insufficient insulin secretion. Failure of blood glucose management in patients with diabetes leads to development of severe complications affecting end organs including kidneys. Many factors have been considered to contribute to the development of type 2 diabetes and its complications such as diet and lifestyle. Recent studies have demonstrated the role of inflammatory process in the progression of diabetes and the etiology of complications. Prevalence of chronic kidney disease (CKD) in the patients with type II diabetes is estimated to range between 30% and 40%. Diabetic nephropathy is a leading causative factor of CKD and end-stage renal disease (ESRD). It is attributed to dysglycemia involving chronic hyperglycemia and acute glycemic fluctuation. The mechanisms underlying the effects of dysglycemia on kidney function are postulated to be the activation of inflammation and increased oxidative stress. Excessive production of reactive oxygen species (ROS) in responses to hyperglycemia, advanced glycosylation end products (AGEs) and cytokines contributes to kidney injury [1], [2], [3]. Intervention of diabetes with anti-inflammatory agents and antioxidant has been demonstrated to reduce risk of CKD [4], [5], [6].

Indoxyl sulfate (IS), a metabolite of tryptophan, accelerates the progression of CKD. It is synthesized in the liver from indole and normally excreted into urine. As kidney impairment, IS accumulates in serum due to impaired renal clearance. Accumulated IS in serum leads to pathological changes in the basolateral membrane of renal proximal tubular cells [7]. IS has been demonstrated to cause excessive production ROS which activates nuclear factor-κB (NF-κB) and p53 [8]. Activation and induction of NF-κB by IS suppress proliferation and induce senescence in proximal tubular cells [9]. In addition, IS induces expression of fibrotic genes such as transforming growth factor-β1 (TGF-β1) and α-smooth muscle actin (α-SMA), as well as inflammatory genes such as monocyte chemotactic protein-1 (MCP-1) in renal cells [9].

The enzyme dipeptidyl peptidase- (DPP-) 4, also known as adenosine deaminase complexing protein 2, degrades both 2 GLP-1 and GIP to their inactive metabolites. Pharmacological competitive inhibition of DPP-4 increases the half-life and bioavailability of active incretins, enhancing their physiological effect. Recent paper proved that augmentation of GLP-1 by inhibition of DPP-4 improved left ventricle performance in response to stress in patients with coronary artery disease [10]. There is evidence suggesting protective effects of GLP-1 on various cardiovascular risk factors [11]. Additionally, a body of evidence suggests that treatment with DPP-4 inhibitor attenuates kidney injury and improves acute and chronic injury [12], [13]. Especially, it has been demonstrated that DPP-4 inhibitors are well tolerated and safe in diabetic patients with renal impairment [14], [15], [16].

In the present study, we investigated the effect of a DPP-4 inhibitor on proximal tubular cells exposed to IS (IS) and to elucidate the underlying mechanism.

## Materials and Methods

### Cell Culture

Human kidney 2 (HK-2) cell is a proximal tubular cell (PTC) line (ATCC CRL-2190) were cultured in DMEM medium supplemented with 2% (vol./vol.) fetal calf serum in the standard fashion. The cells in this experiment are used within three to four passages and are examined to ensure that they demonstrated the specific characteristics of endothelial cells. Cells were incubated overnight prior to treatment. After 1 mM IS stimulation, cells were treated with a serial concentration of DPP-4 inhibitor (Diportin A; Sigma) for 20 hours.

### Cell Viability

Cell viability is determined using 3- (4,5-cimethylthiazol-2-yl)-2,5-diphenyl tetrazolium bromide (MTT) assay. The MTT assay depends on the extent to which viable cells convert MTT bromide to an insoluble colored formazan product that can be determined spectrophotometrically. After treatment, cells are harvested and washed in PBS, and 200 mL of DMEM without phenol red, containing 5 mg/mL MTT, is added to each well. Three hours later, the medium is aspirated, and the converted dye is solubilized with isopropanol (0.1 N HCl in isopropanol). The resulting absorbance from each well is measured at a wavelength of 570 nm with background subtraction at 630 nm.

### Reactive Oxygen Species (ROS) Assay

To evaluate cellular levels of ROS, cells were seeded in 96-well plates and pretreated with IS (1 mM) for 3 h. After the removal of IS from wells, cells were incubated with peroxide-sensitive probe 2′7′-dichlorofluorescein diacetate (DCFDA; Invitrogen) according to the manufacturer’s instructions. The fluorescence intensity (relative fluorescence units) was measured at 485-nm excitation and 530-nm emission by using a fluorescence microplate reader. Cellular oxidative stress was detected using the cell-permeable fluorogenic probe CellROX (Molecular Probes), and the image was captured with a confocal laser-scanning microscope (LSM510, Zeiss) [17].

### Subcellular Fractionation

Cells were washed with PBS and subsequently lyzed with lysis buffer (10 mM HEPES, pH7.5; containing 15 mM KCl, 2 mM MgCl_2_, 0.1 mM EDTA, 1 mM dithiothreitol, and 1 mM PMSF,) for 10 min. Cell lysates were centrifuged at 2,500 g for 10 min at 4°C and the supernatant containing the cytosol was further centrifuged at 20,000 g for 10 min at 4°C. The pellets were washed with PBS, resuspended in nuclear buffer (25 mM HEPES, pH7.5, 1 M KCl, 0.1 mM EDTA and 1 mM PMSF), and centrifuged at 12,000 g for 10 min at 4°C.

### Western Blot Analysis

Cells were lysed using cell lysis buffer (Cell Signaling Technology, Beverly, MA, USA). Sample lysates were subject to SDS-PAGE according to a standard protocol. After being transferred to membranes, the samples were immunoblotted with 1000-fold diluted primary antibodies including phosphorylated Akt (p-Akt), Akt (Akt), α-tubulin, phosphorylated Erk (p-Erk), Erk(Erk), phosphorylated p65, α-SMA, TGF-β and β-actin (Cell signaling, MA,USA). The resulting membranes were incubated with secondary antibodies conjugated to horseradish peroxidase (Santa Cruz Biotechnology, CA, USA). Bands were revealed using an enzyme-linked chemiluminescence detection kit (Amersham Biosciences, Piscataway, NJ, USA) and band density is quantified using an analyser (LumiVision; Aisin, Kariya, Japan).

### Statistical Analyses

The value of each treatment group was presented as a mean with the standard deviation of triplicates. The data were compared using the one way ANOVA with Tukey–Kramer multiple comparisons post-test. The differences were considered significant for *p* values less than 0.05.

## Results

### DPP-4 Inhibitor Ameliorated IS-induced Cytotoxicity in HK-2 Cells

**Figure 1:**
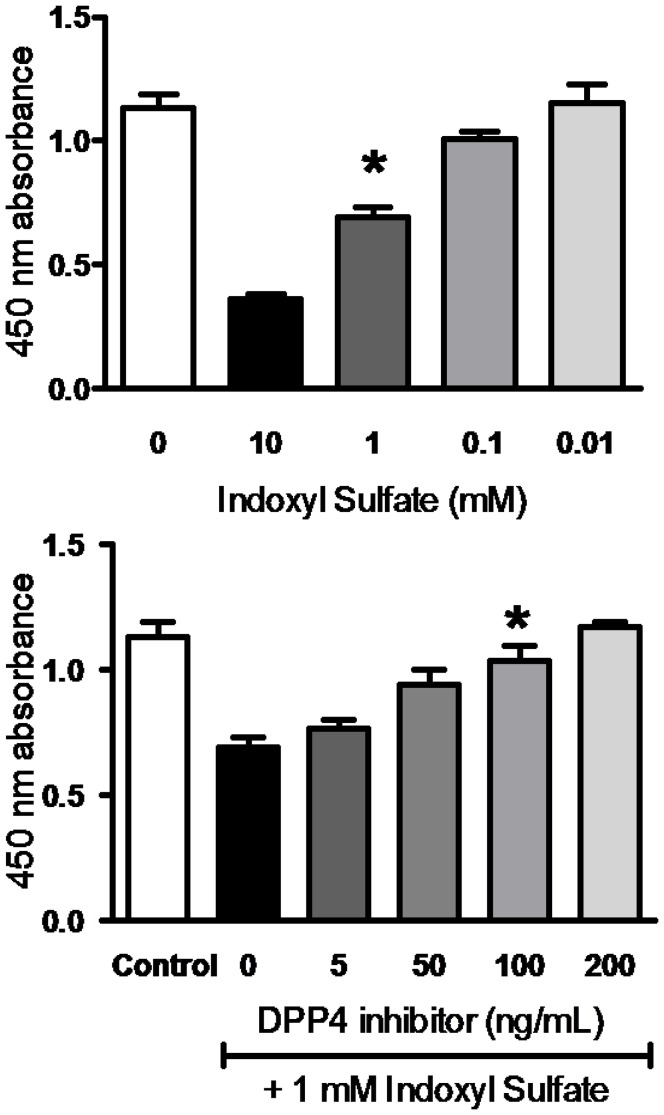
HK-2 viability inhibited by IS and restored by DPP-4 inhibitor. (A) Dose-dependent effect of IS on HK-2 cells viability. HK-2 cells were incubated with various concentration of IS for 3 days. (B) Dose-dependent effect of DPP-4 inhibitor on IS-treated HK-2 cells. HK-2 cells were incubated with 1 mM of IS for 3 days followed by treatment with various concentrations of DPP-4 inhibitor. Cell viability was determined by MTT assay, and data are presented as percentage of viability.

**Figure 2:**
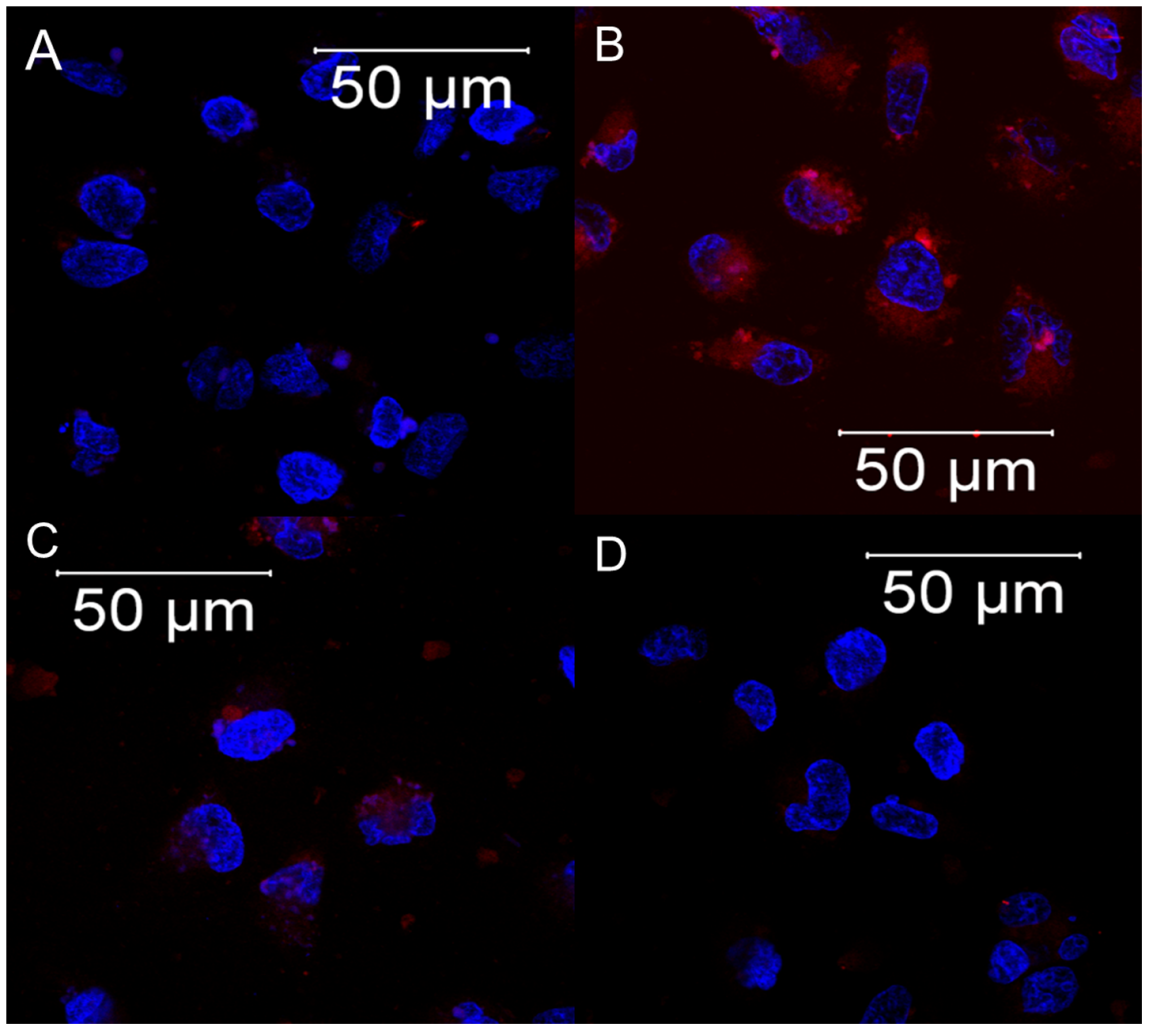
Reactive oxygen species assays. (A) Control (B) 1 mM IS (C) 1 mM IS/100 ng/mL DPP-4 inhibitor (D) 1 mM IS/1 µM probenecid. Cellular oxidative stress was detected using the cell-permeable fluorogenic probe CellROX (Molecular Probes), and the image was captured with a confocal laser scanning microscope (LSM510, Zeiss).

We examined the cytotoxic effect of IS at various concentrations on the viability of HK-2 cells using MTT assay. As shown in Figure 1A, incubation with IS for 3 hr resulted in a significant decreased viability in HK-2 cells in a dose-dependent fashion from 0.01 to 10 mM. Treatment with 1 mM of IS inhibited of cell proliferation to 61.63±7.68%, and thus exposure to 1 mM of IS was employed for following experiments. As exposure to IS arouse oxidative stress to cells, we evaluated the level of ROS production in IS-treated HK-2 cells. The results showed that a significant production of ROS was observed in the HK-2 cell after exposure to 1 mM of IS (Figure 2B). We next determined the effect of DPP-4 inhibitor on viability of HK-2 cells upon IS exposure. Cytotoxic effect of IS on HK-2 cells was significantly attenuated upon DPP-4 inhibitor treatment in dose-dependent manner (Figure 1B). Augmented viability of IS-treated HK-2 cells was restored to 83.38±9.49% and 89.59±7.04% by a treatment with DPP-4 inhibitor at concentrations of 50 and100 ng/mL, respectively. ROS production in IS-treated HK-2 cell was restored by treatment with DPP-4 inhibitor at 100 ng/mL (Figure 2C). In parallel, IS-treated HK-2 cells were resulted in a decrease in ROS production in presence of probenecid (1 µM) which blocks the uptake of IS in HK-2 cells (Figure 2D).

### DPP-4 Inhibitor Suppressed IS-induced Apoptosis through Activation of AKT, and ERK1/2 Pathway in HK-2 Cells

**Figure 3:**
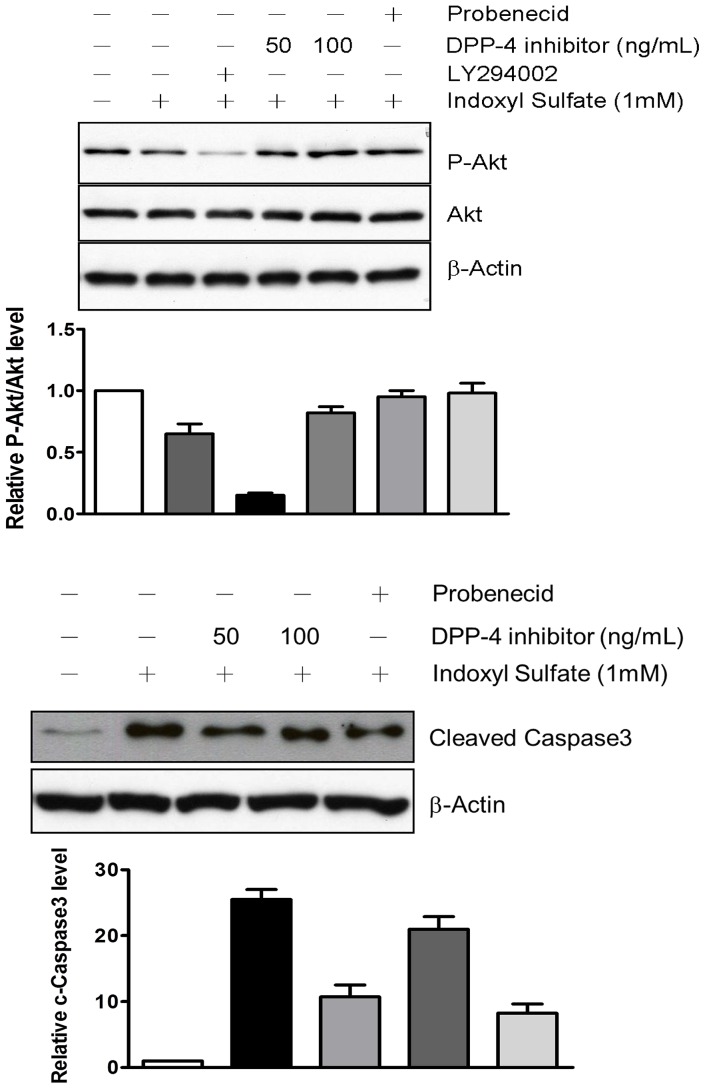
DPP-4 inhibitor reversed the changes in levels of p-AKT, AKT (A) and caspase-3 (B) induced by 1 mM IS. Cells were exposed to 1 mM IS for 3 h and then treated with 100 ng/mL DPP-4 inhibitor. The treated cells were lysed for determination of p-AKT, AKT and Caspase-3 by immunoblot using specific antibodies and chemiluminescence development.

**Figure 4:**
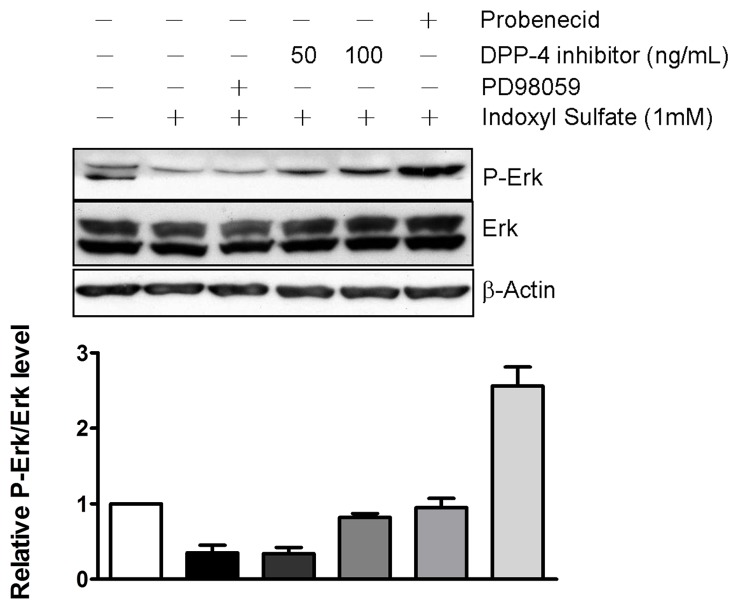
DPP-4 inhibitor restored level of p-ERK induced by 1 mM IS. Cells were exposed to 1 mM IS for 3 h and then treated with 100 ng/mL DPP-4 inhibitor. The treated cells were lysed for determination of p-ERK and ERK by immunoblot using specific antibodies and chemiluminescence development.

We demonstrated that IS induced an increase in intracellular production of ROS in HK-2 cells and that DPP-4 inhibitor restored the cell growth of HK-2 cell upon IS exposure. Upon stimulation by cytokine or IS, intracellular production of ROS lead to an activation of AKT. Accordingly, we investigated the mechanism underlying the protective effect of DDP-4 inhibitor on IS-induced cell death with emphasis on survival singling pathways. We observed that level of phosphorylated AKT was decreased by the exposure to 1 mM of IS (Figure 3A). Treatment of IS-treated HK-2 cells with LY294002, an AKT inhibitor, led to a significant decrease in AKT phosphorylation. Moreover, the level of cleaved caspase 3 was elevated in response to IS exposure (Figure 3B). The suppressed phosphorylation of AKT was significantly reversed in a presence of DPP-4 inhibitor, whereas the level of cleaved caspase 3 was slightly affected. We also determined whether the extracellular signal-regulated kinase (ERK), which fine-tunes the balance between survival and death-promoting genes throughout the MAPK pathway, is involved in the protective effect of DPP-4 inhibitor. Exposure to IS resulted in a decrease in level of phosphorylated ERK. IS-induced reduction of ERK activity was restored by DPP-4 inhibitor in a dose-dependent manner (Figure 4). IS-induced suppressions of phosphorylation of AKT and ERK were also restored in presence of probenecid (1 µM), the OAT inhibitor.

### DPP-4 Inhibitor Reduced the Expressions of Fibrotic Gene in IS-treated HK-2 Cells

**Figure 5:**
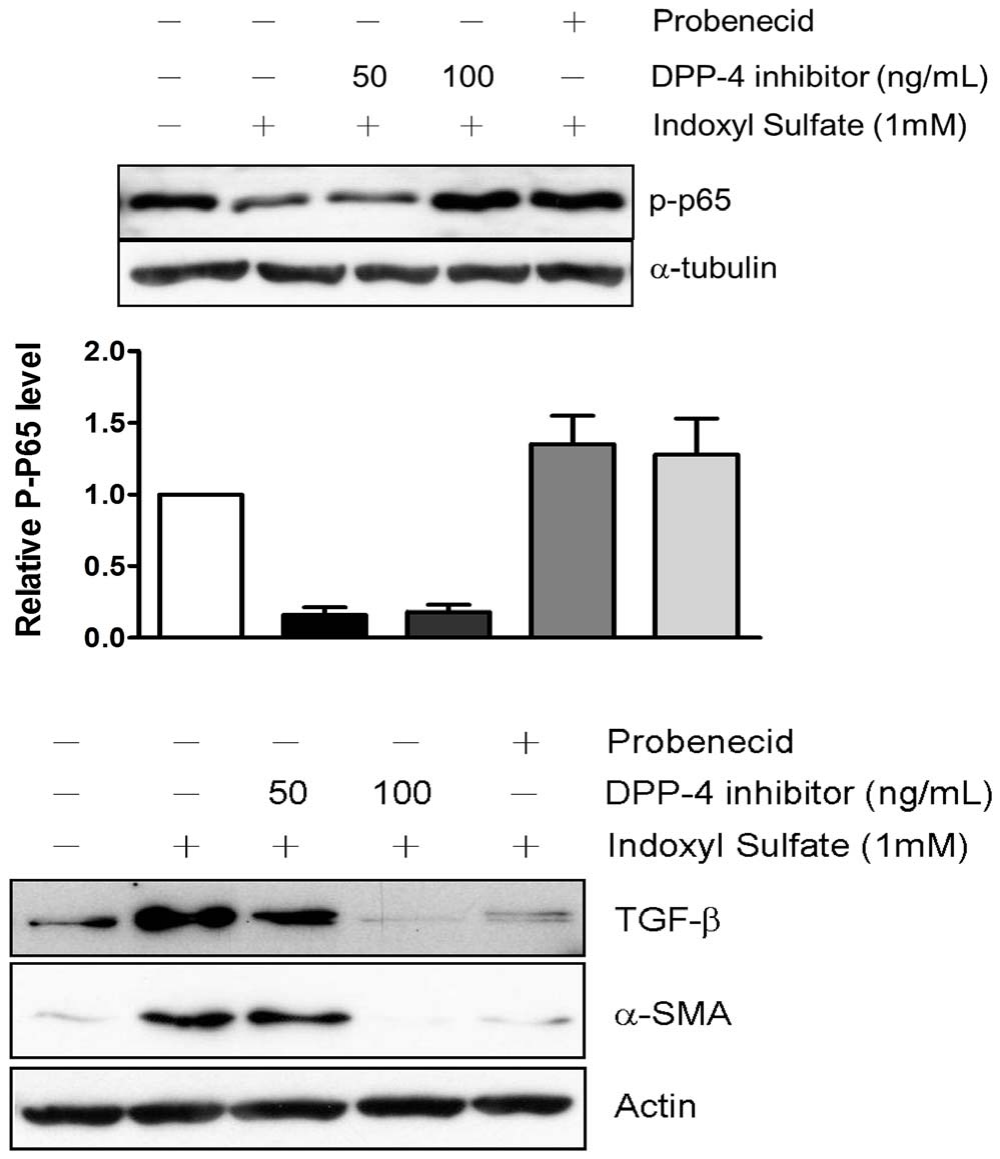
DPP-4 inhibitor reduced the elevated levels of (A) cytosolic NF-κB p65, (B) TGF-β and α-SMA induced by 1 mM IS. (A) cytosolic NF-κB p65; (B) TGF-β and α-SMA. Cells were exposed to 1 mM IS for 3 h and then treated with 100 ng/mL DPP-4 inhibitor. The treated cells were lysed for determination of TGF-β and α-SMA by immunoblot using specific antibodies and chemiluminescence development.

It is evident that IS-mediated ROS production leads to pathological fibrosis that contributes to progression of CKD^18, 19^. NF-κB has been shown to play a pivotal role in IS-mediated ROS generation and inflammatory responses [18], [19]. Our data showed that IS induced NF-κB activation in HK-2 cells in a dose dependent fashion (Figure 5A). It is known that NF-κB promotes TGF-β signaling which plays a critical role in orchestration of fibrotic gene changes. We thus determined the effect of DPP-4 inhibitor on expression of fibrosis in HK-2 cells upon IS stimulation. The results showed that expressions of TGF-β that were upregulated in response to IS stimulation. In consistent with the result, an increased α-SMA expression in IS-treated HK-2 cells was observed. The elevations of TGF-β and α-SMA expression were suppressed significantly by treating with DPP-4 inhibitor in a dose-dependent fashion (Figure 5B). We also observed that probenecid (1 µM) inhibited the activation of NF-κB and elevation of TGF-β and α-SMA levels induced by IS in HK-2 cells.

## Discussion

In the present study, we demonstrate that reno-protective role of DPP-4 during CKD with elevated protein binding protein, indoxyl sulfate. Exposure of proximal tubular cells to IS led to dose-dependent cell death and significant ROS production. Our results showed that IS treatment led to a decreased phosphorylation of AKT and ERK and an activation of caspase-3. DPP-4 inhibitor treatment reversed the changes in levels of p-AKT and caspase-3 as well as p-ERK. We found that IS induced an activation of NF-κB and upregulated TGF-β and α-SMA expressions. The elevated expressions were suppressed by DPP-4 inhibitor.

Indoxyl sulfate, a uremic toxin, is elevated in patients with renal inefficiency [20]. Accumulation of IS in serum induces production of ROS, leading to cell damage and death [8], [21]. Kim et al demonstrated that IS (25 µg/mL) induced apoptotic cell death of rat renal tubular cells through an activation of ERK1/2 and p38 MAPK [22]. In endothelial cell, IS induces apoptosis through p38MAPK activation and downstream NF-κB signaling pathway [23]. A recent study has reported that IS diminish protective effect of erythropoietin on vessel through suppressing erythropoietin-induced AKT phosphorylation [24]. In the present study, we found that IS (1 mM) reduced the phosphorylation of AKT in human proximal tubular cells in an association with changes in level of p-ERK. It is suggested that a cross-talk between the Ras-Raf-MEK-ERK and the PI3K-AKT pathways may occur in proximal tubular cells upon oxidative stress induced by IS, which was restored by DPP-4. Our data also demonstrated that the IS-induced changes in apoptotic signaling pathway were reversed by DPP-4 inhibitor treatment. In agreement with our points, an animal study showed that DPP-4 inhibitor improves functional outcome after renal ischemia-reperfusion injury incorporating with decreased apoptosis [13]. It indicated that DPP-4 inhibitor rescue cell damage by IS through intervening apoptosis signaling pathways including as decreased casepase 3.

NF-κB involved in a broad variety of cellular functions including stress-induced, inflammatory responses and regulator of cell fate decision. Activation of NF-κB signaling pathway plays a critical role in cell biological response to IS challenge. IS stimulates free radical production and subsequently activates NF-κB and its downstream effector proteins [25], [26]. Our results are consistent with the findings of previous study that IS elevates NF-κB nuclear translocation. Along with NF-κB activation, IS has been reported to increase TGF-β expression epithelial cells [27]. Treatment of 5/6-nephrectomized rats with IS has promoted the progression of CKD with enhanced TGF-β expression [28], [29]. Our data are in agreement with previous study that IS induces generation of ROS followed by increased expression of TGF-β, in term of kidney fibrosis. α-SMA is commonly expressed in fibroblastic cells and is responsible for kidney fibrosis upon stimulation such as cytokine or mechanical damage [30], [31]. α-SMA is thought to participate in epithelial-to-mesenchymal transition (EMT), which is critical for tissue remodeling and metastasis [32], [33]. Expression of fibrotic markers, TGF-β and α-SMA were suppressed by DPP-4 inhibitor after IS- Infusion. Our results indicate that DPP-4 inhibitor may have a potential to delay fibrotic progression caused by diabetic nephropathy while it is prescribed to patient with diabetes.

In conclusion, DPP-4 inhibitor, a first-line medication for diabetic nephropathy, reduces the impact of IS through the anti-apoptotic and anti-fibrotic properties.

These studies place the vital cross talk between DPP-4 inhibitor and AKT/ERK at the center of reno-protection in the diabetic kidney. Furthermore, we add new meaning to kidney fibrosis, because for the first time, DPP-4 inhibitor may exert antioxidant to achieve the synergistic beneficial effect on cell survival. Further studies are required to explore the effect of DPP-4 inhibitor and to elucidate the underlying mechanism.
